# Geoepidemiological perspective on COVID-19 pandemic review, an insight into the global impact

**DOI:** 10.3389/fpubh.2023.1242891

**Published:** 2023-10-19

**Authors:** Alexandre Vallée

**Affiliations:** Department of Epidemiology and Public Health, Foch Hospital, Suresnes, France

**Keywords:** COVID-19, SARS-CoV-2, pandemic, geoepidemiology, epidemiology, public health, spatio-temporal epidemiology

## Abstract

The COVID-19 pandemic showed major impacts, on societies worldwide, challenging healthcare systems, economies, and daily life of people. Geoepidemiology, an emerging field that combines geography and epidemiology, has played a vital role in understanding and combatting the spread of the virus. This interdisciplinary approach has provided insights into the spatial patterns, risk factors, and transmission dynamics of the COVID-19 pandemic at different scales, from local communities to global populations. Spatial patterns have revealed variations in incidence rates, with urban-rural divides and regional hotspots playing significant roles. Cross-border transmission has highlighted the importance of travel restrictions and coordinated public health responses. Risk factors such as age, underlying health conditions, socioeconomic factors, occupation, demographics, and behavior have influenced vulnerability and outcomes. Geoepidemiology has also provided insights into the transmissibility and spread of COVID-19, emphasizing the importance of asymptomatic and pre-symptomatic transmission, super-spreading events, and the impact of variants. Geoepidemiology should be vital in understanding and responding to evolving new viral challenges of this and future pandemics.

## Introduction

The COVID-19 pandemic showed a profound impact on societies worldwide, challenging healthcare systems, economies, and daily life (1–3). To effectively understand and combat the spread of pandemic, a multidisciplinary approach that incorporates both epidemiology and geography required (4, 5). This emerging field, known as geoepidemiology, examines the spatial patterns, risk factors, and transmission dynamics of COVID-19, for example, at various scales, from local communities to global populations (6–9). Geoepidemiology combines the power of spatial analysis, data science, and public health to gain insights into how the virus spreads, clusters in specific regions, and affects different populations (10–13). By analyzing the geographic distribution of COVID-19 cases, researchers and public administrations can identify hotspots, understand the influence of environmental factors, and develop specific strategies for prevention and control the pandemic (14, 15).

This interdisciplinary approach has revealed valuable information about the transmissibility and spread of COVID-19 pandemic (16, 17). It has shed light on determinants, such as urban-rural divides, regional hotspots, cross-border transmission, disparities in healthcare access, neighborhood-level variations, seasonal influences, and the role of behavioral patterns in the spread of the virus (18–21). Moreover, studies have explored the impact of risk determinants such as age, underlying health conditions, socioeconomic factors, occupation, demographics, and behavior on the vulnerability and outcomes of individuals and communities (22, 23).

National and international open data have allowed researchers to generate comprehensive insights and inform evidence-based response strategies (24, 25). Lessons learned from successful interventions, as well as failures, have shaped the global response to the pandemic and provided valuable insights for future preparedness efforts (26).

In this context, the exploration of the geoepidemiology aspect of the COVID-19 pandemic allows public policies to enhance more effective strategies to mitigate transmission (27), protect vulnerable populations (28, 29), allocate resources efficiently (30), and ultimately reduce the impact of the COVID-19 pandemic on public health and societies worldwide. Thus, this review focused on the interest and applications of geoepidemiology analyses during the COVID-19 pandemic.

### Search strategy

PubMed Medline database was used for the research, with only articles in English language, using the following terms: “COVID-19,” “geo-epidemiology” and “spatial epidemiology.” Articles included in this review were both, original research, review, viewpoint, and case reports. Literature was searched from inception to 2023.

### Spatial patterns

#### Identification of hotspots and the implementation of targeted control measures to minimize the spread of the virus

Studies have revealed stark spatial variations in COVID-19 incidence and mortality rates (31, 32). Factors such as population density, urbanization (33, 34), and transportation networks have a major role in virus transmission (35, 36). Dense urban areas with high population mobility have often experienced higher infection rates (37, 38). Moreover, regional variations in healthcare infrastructure, socioeconomic conditions, and public health interventions contribute to varying outcomes (39, 40). One prominent spatial pattern observed in the geoepidemiology of COVID-19 pandemic is the contrast between urban and rural areas (41, 42). Urban centers, characterized by high population density, extensive transportation networks, and frequent social interactions, have often experienced higher infection rates compared to rural regions (42). The concentration of cases in urban areas can be attributed to factors such as increased population mobility, crowded living conditions, and higher levels of economic activity (43). COVID-19 pandemic has exhibited the tendency to cluster in specific geographic areas, giving rise to regional hotspots (44, 45). These hotspots can be influenced by several factors, such as international travel hubs, densely populated neighborhoods, and areas with a high prevalence of risk factors such as poverty or comorbidities (37, 46, 47). Thus, identified hotspots around the world remains major to allow authorities to allocate resources, to implement targeted interventions, and to enforce localized lockdown measures to contain the spread (48, 49).

Moreover, peri-urban areas became marginal and degraded with a high rate of criminality, unemployment and in general low level of development indexes (50). Recent investigation have shown that the lockdown's impact on livelihoods was more severe in peri-urban areas than in urban areas (51). This could be consistent with the consensus that the pandemic hit small businesses, daily-wage earners, and low-wage earners, leaving them with no jobs or reduced incomes (52).

#### Highlight the importance of travel restrictions and coordinated public health responses to prevent cross-border transmission

The spread of COVID-19 pandemic has also been influenced by cross-border transmission patterns (53, 54). Proximity to international borders, transportation routes, and global migration patterns have facilitated the introduction and dissemination of the virus across different regions (55). Spatial patterns in COVID-19 cases have revealed disparities in healthcare access (56). Regions with limited healthcare infrastructure, fewer healthcare professionals, and inadequate testing and treatment facilities have often experienced higher case burdens and poorer outcomes (57). These disparities can exacerbate the impact of the virus, particularly in marginalized communities and underserved areas (29, 58, 59). Within cities and towns, the distribution of COVID-19 cases has exhibited neighborhood-level variations (60). Socioeconomic factors, housing conditions, and access to healthcare services can vary significantly across different neighborhoods, leading to differential vulnerability and infection rates (61).

### Risk factors

By analyzing the relationship between COVID-19 pandemic and various risk factors, geoepidemiology has provided valuable insights into the disease's dynamics (62, 63). Studies have shown that age, gender, and underlying health conditions significantly affect vulnerability to infection and disease severity (64, 65). Additionally, socioeconomic factors, such as poverty, overcrowding, and access to healthcare, have been identified as determinants of COVID-19 outcomes (66).

Age has emerged as a significant risk factor in the transmission and severity of COVID-19. Older individuals, particularly those over the age of 65, are more susceptible to severe illness and mortality (67). Age-related physiological changes weakened immune systems, and a higher prevalence of underlying health conditions contribute to increased vulnerability (67). This ensures targeted protection measures for older populations, including prioritized vaccination campaigns and enhanced healthcare support (68, 69).

Socioeconomic determinants have a crucial role in the determination of COVID-19 risk (70). Individuals from lower socioeconomic backgrounds, who may have limited access to healthcare, live in crowded housing, or have occupations that do not allow for remote work, face heightened exposure risks (71, 72). They may also have challenges in accessing testing, treatment, and adhering to preventive measures due to economic constraints.

Certain occupations, such as healthcare workers, frontline workers, and those in essential services, face an increased risk of exposure to COVID-19 due to close contact with infected individuals or the public (73, 74). Workplaces that involve enclosed spaces, limited ventilation, and crowded conditions, such as factories, meatpacking plants, and prisons, have been associated with outbreaks (75–77). Thus, recent studies have highlighted the importance of workplace safety measures, including personal protective equipment (PPE) (78), physical distancing, and regular testing, to mitigate transmission risks (79, 80).

Demographic determinants, such as gender and ethnicity, have been observed to influence COVID-19 outcomes (81, 82). Men have been found to be more susceptible to severe illness and higher mortality rates compared to women (83). Furthermore, certain ethnic and racial groups, including Black, Indigenous, and minority populations, have experienced higher infection rates and worse outcomes due to systemic health disparities, socioeconomic factors, and structural inequities (84).

Individual behaviors have significant roles in COVID-19 transmission (85, 86). Determinants such as adherence to preventive measures (mask-wearing, hand hygiene, physical distancing), participation in social gatherings, travel, and compliance with public health guidelines influence the risk of COVID-19 infection and transmission (87–89). This knowledge can inform targeted public health messaging, community engagement strategies, and interventions to promote safe behaviors.

The COVID-19 pandemic has had far-reaching effects on mental health, livelihoods, and food security, pushing many individuals and families into poverty (90, 91). As the pandemic unfolded, it brought about a surge in stress, anxiety, and depression among people worldwide. The uncertainty surrounding the virus, fear of infection, isolation due to lockdowns, and loss of loved ones have taken a toll on mental wellbeing (92).

At the same time, the pandemic led to widespread job losses, reduced work hours, and business closures, particularly in sectors heavily affected by restrictions and social distancing measures. Many people found themselves without income or faced significant reductions in earnings, making it challenging to meet basic needs and maintain their livelihoods (93).

As the economic situation worsened, food security became a major concern. Disruptions in supply chains, rising food prices, and limited access to food resources exacerbated existing vulnerabilities, especially among low-income and marginalized populations (93).

The combination of mental health challenges, economic hardships, and food insecurity has forced many individuals and families into poverty (94). People living on the brink of poverty before the pandemic found themselves pushed over the edge due to the loss of income and access to essential resources (95).

The impact has been particularly severe in developing countries and marginalized communities, where the social safety net may be inadequate to address the growing needs (96). Vulnerable populations have been disproportionately affected, as they lack job security, social protection, and access to healthcare (97). Thus, addressing the mental health implications of the pandemic is crucial to mitigating its impact on livelihoods and food security. Providing mental health support and resources can help individuals cope with stress and anxiety, enabling them to better navigate the challenges posed by the pandemic.

### Transmissibility and spread

Geoepidemiological investigations have shed light on the transmission dynamics of COVID-19 pandemic, elucidating how the virus spreads within and between communities (98, 99). By examining patterns of mobility, social interactions, and travel, researchers have quantified the impact of human behavior on virus transmission (100, 101). Furthermore, the integration of geographic information systems (GIS) and mathematical modeling has facilitated the prediction of future outbreaks and the evaluation of the effectiveness of control measures (62, 102). These insights have been instrumental in guiding public health responses and policy decisions (5, 103).

COVID-19 pandemic can be transmitted by individuals who are either asymptomatic (showing no symptoms throughout the infection) or pre-symptomatic (infected but not yet showing symptoms) (104). This characteristic of the virus poses challenges in identifying and containing transmission chains. This type of research has highlighted the significance of widespread testing, contact tracing, and quarantine measures to control the spread, even among individuals who do not exhibit symptoms (105).

COVID-19 pandemic has exhibited a pattern of super-spreading events, where a small number of individuals infect a disproportionately large number of people (106, 107). These events typically occur in crowded settings, such as social gatherings, religious ceremonies, and mass gatherings, where close contact and inadequate preventive measures facilitate rapid transmission (108). Thus, underscoring the importance of avoiding large gatherings is major for implementing targeted interventions in high-risk settings.

Travel restrictions, quarantine measures, and testing protocols have been implemented to mitigate travel-related spread (109). Additionally, contact tracing of individuals with travel history has played a crucial role in identifying and containing clusters of cases (110).

The emergence and spread of SARS-CoV-2 variants have further influenced the transmissibility and spread of COVID-19 (111). Variants with increased transmissibility, such as the Alpha, Beta, Gamma, and Delta variants, have led to more rapid spread in certain regions (112). Recent studies have monitored the circulation and impact of these variants, highlighting the need for genomic surveillance, early detection, and adaptive public health strategies to address emerging viral evolution (113, 114).

The spatio-temporal tracking of changed COVID dynamics based on genomic analysis is a cutting-edge approach in understanding the evolving nature of the SARS-CoV-2 virus (115). By analyzing the genetic sequences of the virus collected from different regions and at different time points, scientists can identify genetic mutations and variations that occur over time (116). This allows them to track the spread and transmission patterns of specific viral lineages across geographic areas. Moreover, the identification of new variants and their prevalence in different regions helps to assess their potential impact on disease severity, transmissibility, and vaccine effectiveness (117). This valuable information aids public health authorities in tailoring targeted interventions, optimizing testing strategies, and guiding vaccination efforts to effectively control and respond to the changing dynamics of the COVID-19 pandemic. The spatio-temporal genomic analysis could become an indispensable tool in the global effort to combat the pandemic and mitigate its effects on communities worldwide (118, 119). Nevertheless, this type of analysis needs to access of open and comprehensive data (120).

### Countries disparities, the specific case of African countries

During the initial stages of the pandemic, the number of COVID-19 cases in African countries closely correlated with the volume of international flights into the continent. South Africa and Egypt, with their busiest international airports, experienced the highest case numbers (121). Conversely, countries with limited business and tourism connections to other continents reported lower case numbers.

A significant contributing factor to the relatively lower-case numbers in Africa was the early closure of airports in many countries, particularly those with prior experience in managing epidemic infectious diseases such as Ebola, Tuberculosis, and Lassa fever (122). These nations-initiated disease surveillance and contact tracing at airports much earlier than many other countries, thereby limiting the introduction of COVID-19 cases into the continent. Consequently, the “seeding” of cases was restrained, leading to a delayed onset and slower growth of infections, effectively “flattening the curve” in many African countries (123).

Testing capacity played a crucial role in shaping the reported case numbers. The top testing countries, e.g., USA and Europe, globally conducted significantly higher numbers of RT-PCR tests compared to African nations (57, 124). Limited testing resources, high costs, and the scarcity of necessary equipment and trained personnel hampered extensive testing in Africa. As a result, many cases might have been missed, and some antibody testing suggested that a substantial portion of the population may have already contracted and recovered from the disease (57). Furthermore, the absence of widespread testing might have resulted in some deaths being erroneously attributed to other causes when they were, in fact, due to COVID-19.

The lower population density in Africa compared to other continents played a favorable role in slowing the spread of the virus (125). Rural and widely dispersed communities contributed to limiting the rapid transmission of the disease, as observed in USA (126).

The youthfulness of the African population was also a protective factor. With a less median age in Sub-Saharan Africa compared to Europe, younger individuals were four times less likely to acquire severe symptoms or fatalities from COVID-19 (127). Conversely, countries with slightly older populations, like Egypt and South Africa, had higher case numbers and greater case fatality rates, partially due to higher prevalence of comorbidities associated with severe illness and mortality (128).

Exposure to previous infections, including other coronaviruses and endemic diseases like malaria, tuberculosis, and HIV, may have conferred relative immunity, and contributed to milder COVID-19 presentations in Africa (129, 130). Countries with higher Human Development Index tended to have higher case numbers and worse outcomes, suggesting that healthcare development and access influenced the pandemic's impact in countries (131).

### Global collaboration and lessons learned

Recently it has been highlighted the importance of global collaboration in responding to a pandemic (132, 133). Through the sharing of data, expertise, and best practices, researchers and policymakers have been able to develop a more comprehensive understanding of the COVID-19 pandemic (134). Moreover, investigations have shown the need for improved surveillance systems, data harmonization, and standardized reporting methods for effective global monitoring and response (135).

The complexity of the COVID-19 pandemic has underscored the need for multidisciplinary collaboration (136) (Table 1). Thus, geoepidemiology brings together experts from various fields, including epidemiology, geography, data science, and public health, to analyze spatial patterns, risk factors, and transmission dynamics (137). By integrating diverse perspectives and expertise, researchers have been able to generate comprehensive insights, develop innovative methodologies, and formulate evidence-based strategies to combat the pandemic.

**Table 1:** Contextual parameters potentially influencing COVID-19 transmission.

**Parameters**	**Elements**
Population density	Inhabitants per km^2^
	Inhabitants per household
	Indoor space per person
Social demography	Age population
	Household composition
	Mixing patterns
	Social events
Social practices	Social contacts
	Handwashing, water and sanitation
	Ventilation / air conditioning
Geography	Climate
	Urbanization rate
	Air traffic intensity
	Population movements
	Road networks
Immunity	Prior exposure
	Non-specific immunity
Genomics analysis	Spatio-temporal analysis
	ACE variability
	Genomic surveillance

The COVID-19 pandemic has emphasized the critical role of rapid information dissemination in effective response efforts (138). Studies have relied on real-time data sharing, open-access publications, and online platforms to disseminate findings promptly (139). Rapid dissemination of accurate information is crucial for guiding public health decision-making, enabling policymakers and healthcare professionals to implement evidence-based interventions, and fostering public trust and compliance with preventive measures (140, 141).

The development and distribution of vaccines against COVID-19 have shown the importance of global solidarity and equitable access (142). Global collaboration has emphasized the need for fair vaccine distribution, support for low-income countries, and collaborative efforts to ensure that no one is left behind in the global vaccination campaign (143).

## Conclusion

In conclusion, this study can statute that geoepidemiology has provided invaluable insights into the global impact of the COVID-19 pandemic. By analyzing spatial patterns, risk factors, and transmission dynamics, this interdisciplinary field has contributed to the development of targeted interventions, efficient resource allocation, and informed decision-making. As the pandemic may continue to evolve, geoepidemiological research will have a major role in understanding and responding to these new emerging challenges. Moreover, geo-epidemiology cannot be a stand-alone field but should seek to work with other disciplines and fields in the delivery of its mandate and contribution to infectious disease epidemiology.

## Author contributions

AV: conceptualization, formal analysis, and writing—original draft preparation.
